# Ultra-Low Power Hand Gesture Sensor Using Electrostatic Induction

**DOI:** 10.3390/s21248268

**Published:** 2021-12-10

**Authors:** Hiroshi Fuketa

**Affiliations:** 1 AI Chip Design Open Innovation Laboratory, National Institute of Advanced Industrial Science and Technology (AIST), Ibaraki 305-8568, Japan; h-fuketa@aist.go.jp; 2 Device Technology Research Institute, National Institute of Advanced Industrial Science and Technology (AIST), Ibaraki 305-8568, Japan

**Keywords:** CMOS, electrostatic induction, electret, hand gesture sensor, ultra-low power

## Abstract

This paper presents an ultra-low power hand gesture sensor using electrostatic induction for mobile devices. Two electrodes, which consist of electret foils stacked on metal sheets, are used to recognize two gestures such as hand movements from left to right and right to left. The hand gesture recognition is realized by detecting the electrostatic induction currents induced by hand movements. However, the electrostatic induction currents are significantly small; hence, a hand gesture recognition chip is first designed in this study to amplify and detect the small electrostatic induction currents with low power. This chip is fabricated in a commercial 180 nm complementary metal oxide semiconductor (CMOS) process, and the measurement results indicate that the fabricated gesture recognition chip consumes 406 nW, which is less than 1/100th of the power dissipation of conventional gesture sensors.

## 1. Introduction

User interfaces using hand gestures are promising, because they provide users with more convenient controllability than touch interfaces, which are widely used in mobile devices, such as smartphones and smart speakers. One of methods for recognizing hand gestures is to put sensor devices, such as glove-type [1,2] and ring-type [3] devices, on hands. These wearable gesture sensors can recognize various complicated hand gestures, whereas the users must put the sensor devices on their hand, which is less convenient. Therefore, various remote gesture sensing techniques without any wearable devices have been proposed [4,5,6,7]. The gesture sensing technique based on an image sensor (camera) [4] is one of the most reliable methods for recognizing hand gestures. It can offer gesture recognition over a long distance and detect complicated gestures [4]. However, image sensors and image processing consume large amounts of power, and bulky components such as lenses are required. As another gesture sensing technique, Han et al. demonstrated a photo sensor-based gesture sensor [5]. In this sensor, LED emits light, and the photo sensor detects the reflected light from the hand to recognize the gesture. Although this sensor does not require a lens, the LED and photo sensor consume significant power. Another way to recognize gestures is based on capacitive sensors, which detect small changes in capacitance induced by hand motion [6]. This sensor does not require a lens either; however, the power dissipation is large because an electric field must be generated to detect the change in capacitance. To reduce the power, a gesture sensor using infrared rays was proposed in [7]. Although the power dissipation of this sensor is smaller compared to those of the abovementioned sensors [4,5,6], it is still large because complicated signal processing is required to recognize gestures from infrared rays emitted from the human hand. Therefore, further power reduction is required to implement gesture sensors in battery-operated devices.

**Figure 1:**
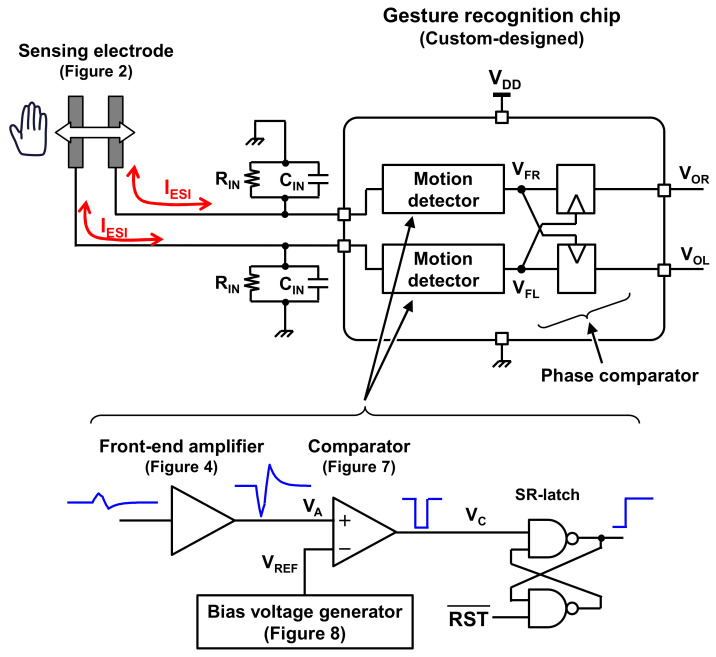
Overview of the proposed hand gesture sensor system. This system consists of sensing electrode using an electret (Figure 2) and a gesture recognition chip, which is first designed in this study to achieve ultra-low power gesture sensing.

**Figure 2:**
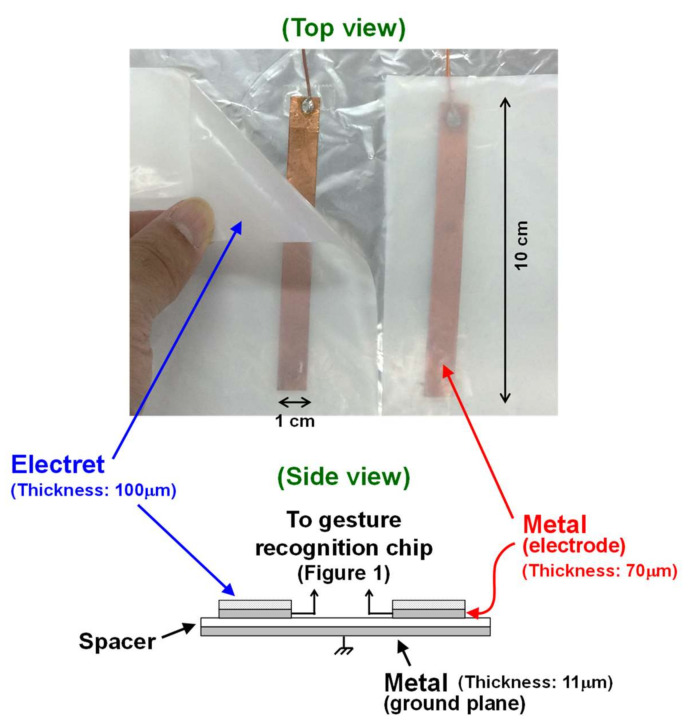
Photograph and cross-sectional view of the sensing electrodes used in this study. Two electrodes are used to recognize hand gesture. Each electrode consists of an electret foil stacked on a metal sheet.

In this paper, an ultra-low power hand gesture sensor using electrostatic induction (ESI) is proposed. Figure 1 shows an overview of the proposed sensor. The proposed gesture sensor is based on an ESI-based motion sensor [8,9]. The electrode consists of a metal sheet and an electret foil, as illustrated in Figure 2. When the hand moves near the electrode, an electric current (*I_ESI_* in Figure 1) is generated by the ESI. By observing the ESI current, hand movements can be detected. In conventional works [8,9], a single electrode was used to detect hand motion. In contrast, two electrodes are utilized in this study, as shown in Figure 2, to recognize hand gestures, such as hand movement from right to left and left to right. The ESI currents generated in the electrodes are significantly small, less than 1 nA [9]. To process such a small current with low power, a sensor front-end circuit, such as an amplifier, comparator, and phase comparator is newly designed in this study, as shown in Figure 1. The measurement results indicate that the proposed sensor consumes 406 nW, which is less than 1/100th of the power dissipation of conventional sensors.

As explained above, the proposed sensor is based on the ESI-based motion detection mechanism proposed in conventional works [8,9]. The circuit proposed in this paper is improved compared to that in [8,9]. That is, the circuit demonstrated in [8,9] requires differential inputs, which are sensitive to manufacturing variability; hence, the yield may deteriorate, whereas the circuit proposed in this work is a single-ended design. Additionally, the intermediate voltage (half of the supply voltage) must be supplied in the conventional circuit [8,9], whereas the proposed circuit operates at a single supply voltage of 3.3 V. A hand gesture sensor based on ESI was demonstrated in [10,11]. Eight gestures can be recognized using the four electrodes in [11]. The conventional sensors can detect more gestures than the sensor proposed in this study, whereas they are not intended to be implemented in mobile devices; hence, the power consumed to recognize hand gestures is not considered. Another technique to detect hand motion using electrostatic induction is proposed in [12,13]. These motion sensors use polydimethylsiloxane (PDMS) films with multiple electrodes, and the voltage waveforms induced by hand motion on the electrodes are acquired by an oscilloscope and processed by a laptop PC to recognize hand gestures [13]. However, the power consumed in the oscilloscope and PC is not considered as well.

In the proposed gesture recognition sensor, a PTFE foil is used as an electret and a cupper sheet is used as metal electrode (the details are explained in Section 4), as shown in Figure 2. These materials are commercially available, and hence fabrication of the sensing electrode is not discussed in this paper. In contrast, the chip to recognize hand gestures, which is denoted by gesture recognition chip in Figure 1, is newly designed in this work. This chip enables ultra-low power gesture recognition, which is the most advantageous point compared to conventional gesture sensors. This feature is especially important for battery-operated devices, since low-power operation can reduce the frequency of battery exchange or recharge.

Therefore, the target applications of the proposed sensor are gesture sensing on battery-operated devices, for example, (1) touchless switches for hygiene purposes and for situations where hands cannot be used freely, such as soap dispenser [14], remote light switch [8], and controller for music player [15]; and (2) mobile devices requiring user interaction, such as laptop PCs, tablets, and smart speakers [14]. The size and thickness of the electrodes used in the proposed sensor are 10 cm^2^ and 200 μm, which is small enough for such devices. In PCs and tablets, for example, the electrodes are placed in both sides of the screen. The gesture recognition chip proposed in this paper can be implemented with the microcontroller of the target devices as a companion chip or can be implemented even in the microcontroller, since the actual silicon area of the gesture recognition chip is significantly small (the details are explained later in Section 4).

The remainder of this paper is organized as follows: The mechanism of the proposed gesture sensor is discussed in Section 2. Section 3 describes the details of the circuit for recognizing hand gestures proposed in this study. The proposed circuit is fabricated in 180 nm complementary metal oxide semiconductor (CMOS) process. The measurement results of the chip are presented in Section 4. Finally, Section 5 concludes the paper.

## 2. Mechanism of the Proposed Gesture Sensor

Here, the mechanism of the ESI-based gesture sensing proposed in this paper is discussed. When the hand moves horizontally over the electrode with an electret, the ESI current (*I_ESI_* in Figure 1) is generated. A previous study [9] proposed that the ESI current *I_ESI_* can be modeled as follows:

(1) $$I_{ESI}=-\frac{Dv_{x}}{h_{e}+d_{e}}h_{e}\sigma ,$$

where D is the depth of the overlap area between the electrode and the hand, $v_{x}$ is the velocity of hand movement, and σ is the charge density of the electret. $h_{e}$ and $d_{e}$ are defined as follows:

(2) $$h_{e}=h/\varepsilon_{e},$$

(3) $$d_{e}=d/\varepsilon_{a},$$

where h is the thickness of the electret, d is the distance between the electret and human body, $\varepsilon_{e}$ and $\varepsilon_{a}$ are relative permittivity of the electret and air, respectively. This means that the hand motion can be detected by observing *I_ESI_*.

**Figure 3:**
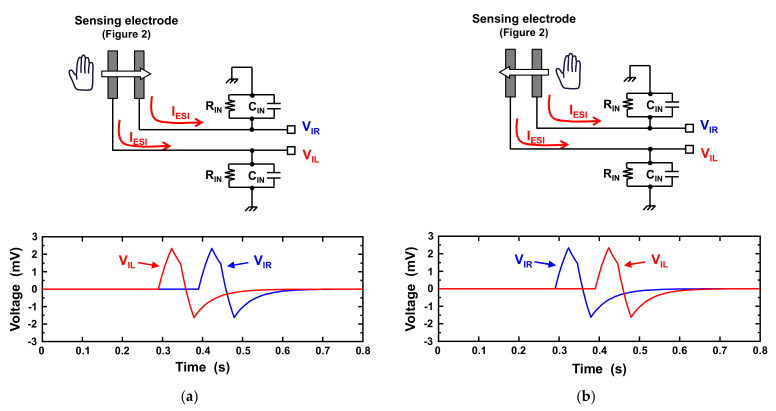
Mechanism of the proposed ESI-based gesture recognition technique. In the proposed technique, two electrodes with an electret are placed at a certain distance from each other. When the hand moves horizontally over the electrode, the ESI current *I_ESI_* is generated, which induces the voltage change in *V*_IL_ and *V*_IR_. (**a**) When the hand moves from left to right, *V*_IL_ changes earlier than *V*_IR_. (**b**) When the hand moves from right to left, V_IR_ changes earlier than *V*_IL_. This means that gestures can be recognized by observing the ESI currents (*I_ESI_*) induced in the two electrodes.

In this paper, gesture recognition based on the ESI-based motion sensing is proposed using multiple electrodes. Figure 3 illustrates the mechanism of the proposed gesture recognition technique. As shown in this figure, a circuit simulation using the ESI current model (1) is conducted. As illustrated, two electrodes with an electret are placed at a certain distance from each other. When the hand moves from left to right, the ESI current *I_ESI_* is first induced in the left electrode, and then that in the right electrode is induced (*V*_IL_ induced by *I_ESI_* in the left electrode changes earlier than *V*_IR_ induced by *I_ESI_* in the right electrode, as shown in Figure 3a). However, when the hand moves from right to left, the ESI current *I_ESI_* is first induced in the right electrode, and then that in the left electrode is induced (*V*_IR_ induced by *I_ESI_* in the right electrode changes earlier than *V*_IL_ induced by *I_ESI_* in the left electrode, as shown in Figure 3b). This indicates that hand gestures (left to right and right to left) can be recognized by observing the ESI currents induced in the two electrodes.

The previous study [9] reported that the ESI current is less than 1 nA. To detect such a small current with low power, we propose an ultra-low power amplifier and a comparator with a bias voltage generator, which are optimally designed for ESI-based gesture sensing in this paper. The details of the circuit implementation are discussed in the next section.

## 3. Circuit Structure

An overview of the gesture recognition chip proposed in this paper is shown in Figure 1. The chip consists of a front-end amplifier, comparator, and reference voltage generator. The details are described in this section.

### 3.1. Front-End Amplifier

**Figure 4:**
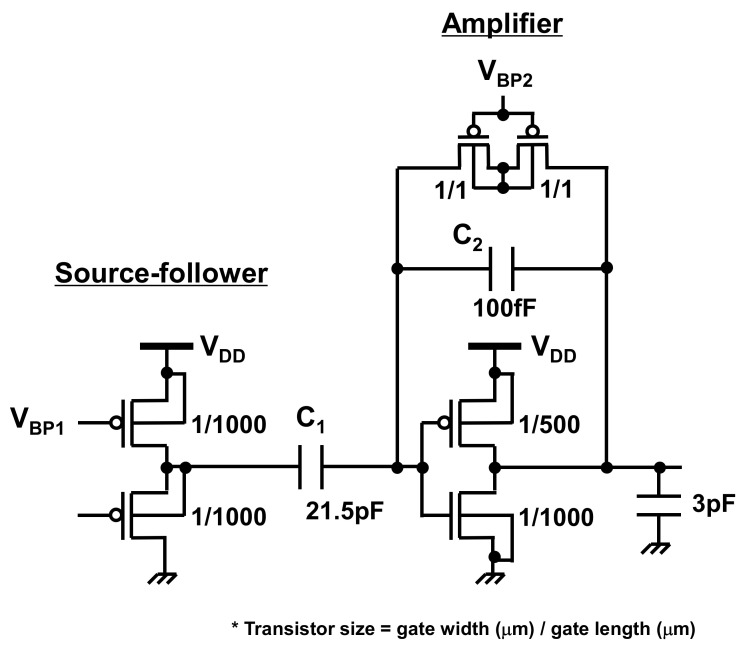
Circuit schematic of the front-end amplifier used in the proposed gesture recognition chip (Figure 1). The front-end amplifier consists of a source follower with an inverter-based amplifier.

**Figure 5:**
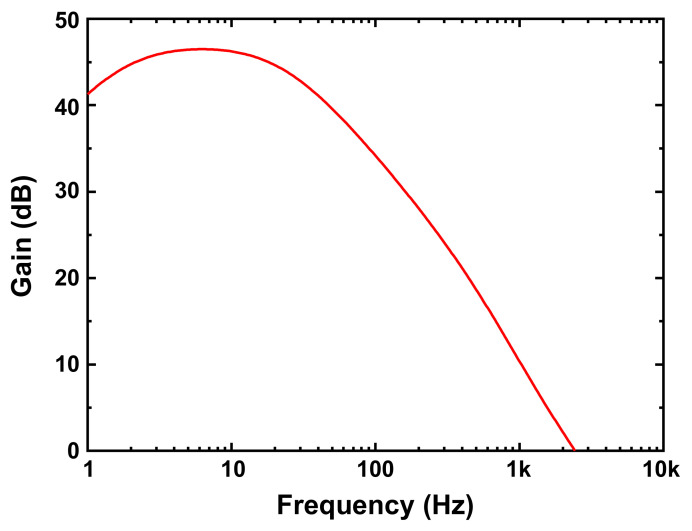
AC characteristics of the front-end amplifier shown in Figure 4.

Figure 4 illustrates the circuit schematic of the front-end amplifier used in the proposed gesture recognition chip. The front-end amplifier consists of a source follower, followed by an inverter-based amplifier. As described in Section 1, a single-ended amplifier is used to avoid mismatch issues in the differential counterpart. The source-follower and inverter-based amplifier are capacitively coupled, and the gain of the amplifier is determined by the ratio of the coupling capacitance (C_1_) to the feedback capacitance (C_2_). The simulated AC characteristics of the proposed front-end amplifier are shown in Figure 5. The frequency of the signal induced by the hand gesture is approximately 10 Hz, as depicted in Figure 3. The gain of the amplifier reaches a maximum value of 46 dB (~C_1_/C_2_) at 10 Hz. The bias voltages, *V*_BP1_ and *V*_BP2_, are generated by the bias voltage generator, which is explained in Section 3.2.

**Figure 6:**
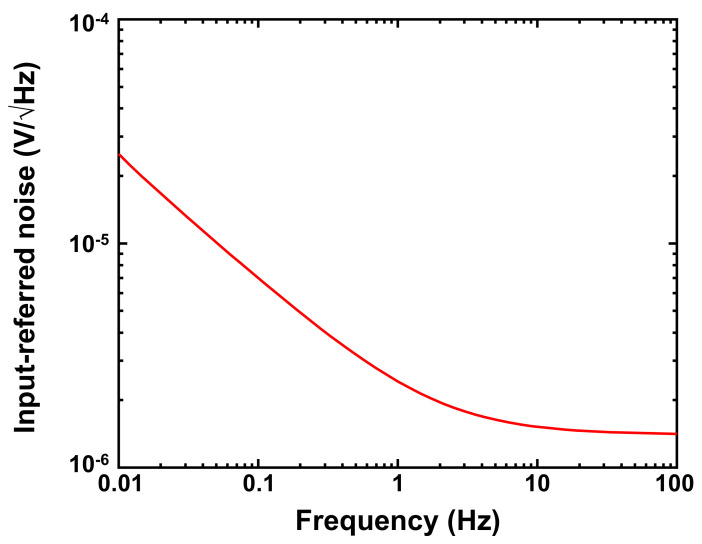
Simulated input-referred noise of the front-end amplifier shown in Figure 4.

Figure 6 shows the simulated input-referred noise of the front-end amplifier as a function of frequency. The input-referred noise in the frequency range of 0.01–100 Hz is 15 μV_rms_ (output noise is 1.84 mV_rms_), which is similar to that of the amplifier used in the conventional ESI-based motion sensor [9]. The effect of noise induced by the front-end amplifier is discussed in Section 4.

### 3.2. Comparator with a Bias Voltage Generator

**Figure 7:**
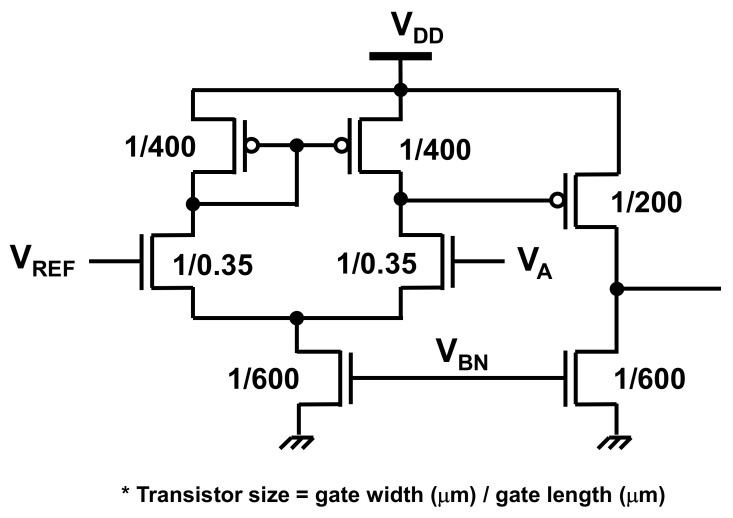
Circuit schematic of the comparator used to detect hand motion. *V*_A_ is the amplified signal induced by the hand movement, as illustrated in Figure 1.

After the input signal is amplified in the front-end amplifier, it is compared with a certain voltage (*V*_REF_ in Figure 1) to detect the hand motion. When the voltage of the signal becomes lower than *V*_REF_, the hand motion is detected. A schematic of the comparator is shown in Figure 7.

**Figure 8:**
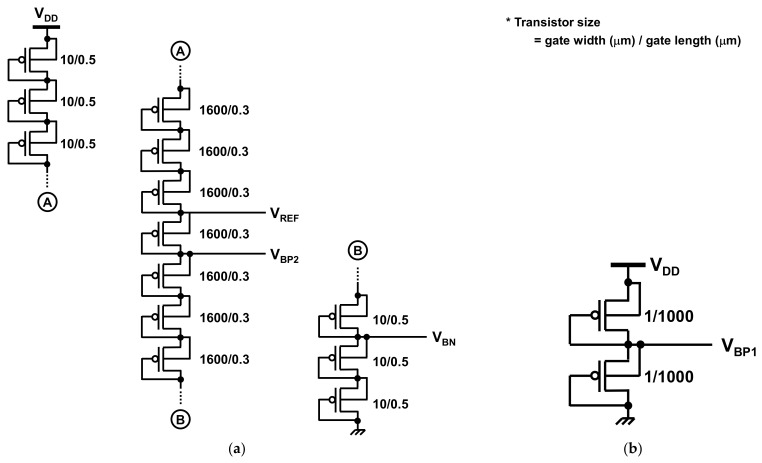
(**a**,**b**) Circuit schematic of the bias voltage generator. This circuit generates the four bias voltages; *V*_BP1_ and *V*_BP2_ are used in the front-end amplifier (Figure 4), whereas *V*_REF_ and *V*_BN_ are used in the comparator (Figure 7).

The front-end amplifiers and comparator require bias voltages (*V*_BP1_ and *V*_BP2_ in Figure 4, as well as V_BN_ in Figure 7). Figure 8 shows the circuit schematic of the bias voltage generator, which generates the bias voltages as well as *V*_REF_. To generate *V*_BN_, *V*_BP2_, and *V*_REF_, the supply voltage is divided using diode-connected transistors, as shown in Figure 8a. The bias voltage of the source follower, *V*_BP1_, in Figure 4 is generated by the replica source follower whose input and output are shortened, as illustrated in Figure 8b.

## 4. Measurement Results

**Figure 9:**
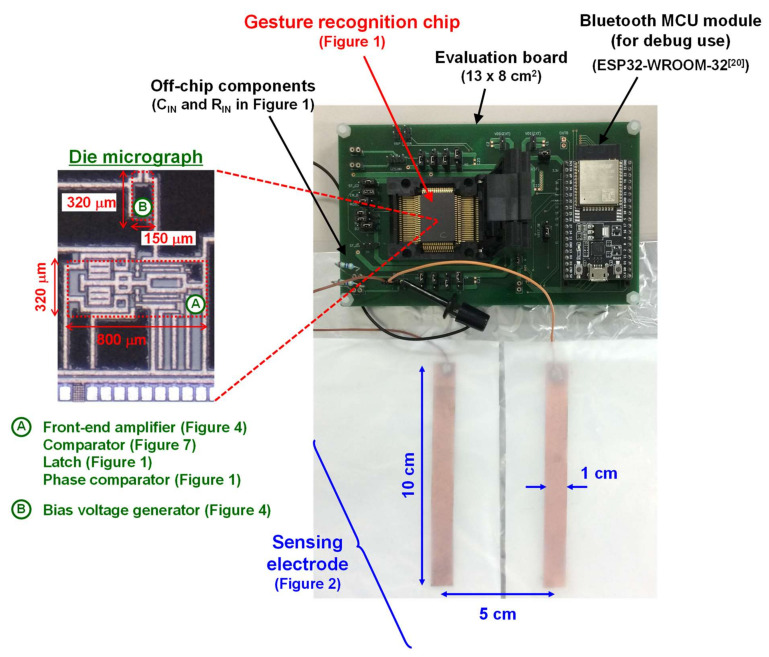
Measurement setup of the proposed hand gesture recognition system (Figure 1). The chip to recognize gesture with ultra-low power was first designed and fabricated in the 180 nm CMOS process. Its die photograph is also depicted. The two electrodes were placed 5 cm apart from each other. Each electrode consists of a PTFE foil as an electret stacked on copper sheet (Figure 2).

**Figure 10:**
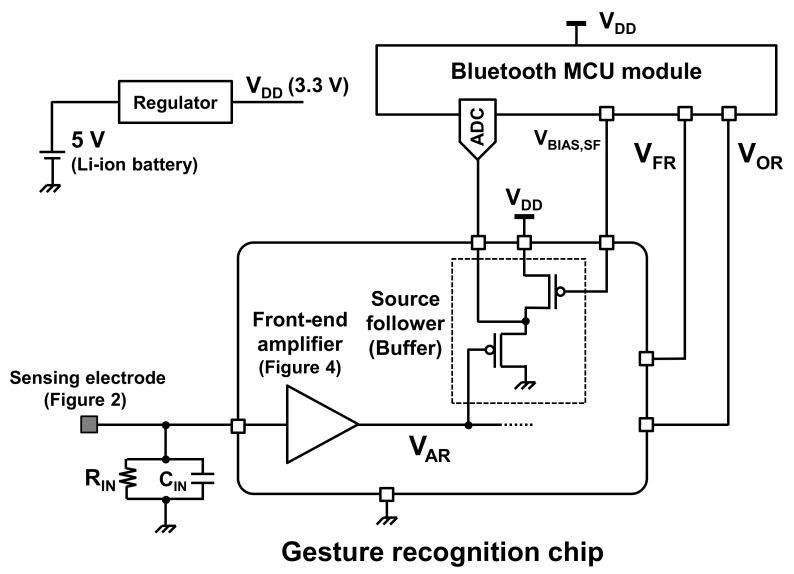
Details of measurement setup in Figure 9. The waveforms are acquired by a battery-operated Bluetooth microcontroller module, and they are transmitted via Bluetooth. The acquired waveforms are shown in Figure 11. Please note that this Bluetooth module is only used for debug purpose and data transmission via Bluetooth is not required in practical use case.

The measurement setup is shown in Figure 9. Details of the setup are explained in Figure 10. The gesture recognition chip was fabricated in the commercial 180 nm CMOS process. The proposed circuit explained in Section 3 occupies 0.3 mm^2^. The chip was sealed in a ceramic package (QFP80) and mounted on an evaluation board, as shown in Figure 9. The input resistance R_IN_ and capacitance C_IN_ (in Figure 1) are 4.7 MΩ and 10 nF, respectively. The two copper electrodes (TERAOKA copper foil conductive adhesive tape No. 8323 [16]) were placed 5 cm apart from each other. The size and thickness of each electrode were 1 × 10 cm^2^ and 70 μm, respectively. A commercially available 100 μm-thick PTFE sheet (NICHIAS TOMBO^TM^ No. 9001 Naflon^®^ PTFE tape [17]) rubbed by paper is used as an electret [8,9,18] and placed on the copper electrode, as shown in Figure 2. The charge density of the electret (σ in (1)) was estimated to be −40 μC/m^2^ [9]. In practical use case, the PTFE sheet should be charged by corona charging to be used as an electret [19]. Gerlach et al. reported that such corona-charged electrets can keep their charge for more than 1000 days at 95 °C, and 50 years at room temperature and low relative humidity [19].

**Figure 11:**
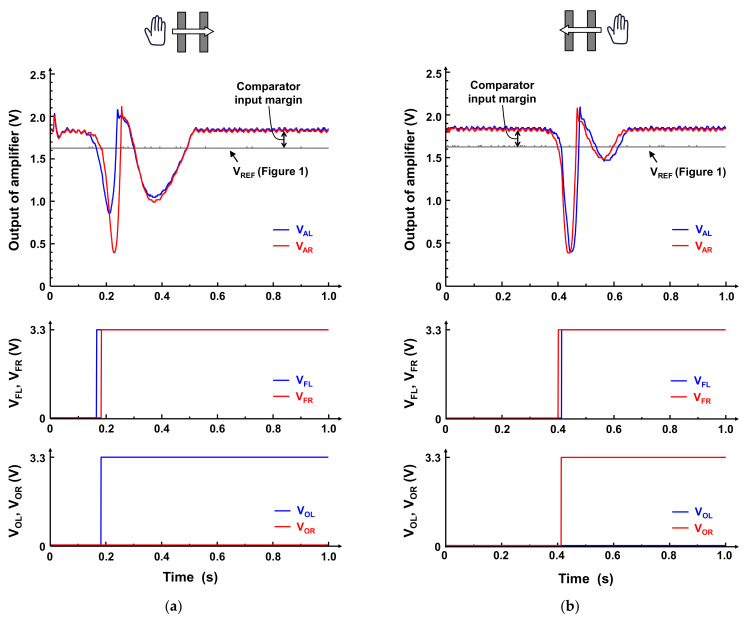
Measured waveforms when the hand moves horizontally on the electrode with a distance of 5 cm between the hand and the electrode (**a**) from left to right and (**b**) from right to left. V_AL_ and V_AR_ denote the amplified signals (V_A_ in Figure 1) input from left and right electrodes, respectively. V_FL_ and V_FR_ are latched output of the comparator, and V_OL_ and V_OR_ is the outputs of the proposed gesture recognition chip, as shown in Figure 1. By observing V_OL_ and V_OR_, the gestures, such as the hand movement from left to right (V_OL_ goes high) and from right to left (V_OR_ goes high), can be recognized.

Figure 11 shows the measured waveforms when the hand moves from left to right (Figure 11a) and from right to left (Figure 11b) horizontally on the electrode with a distance of 5 cm between the hand and the electrode. These waveforms are obtained by a battery-operated Bluetooth microcontroller module (ESP32-WROOM-32 [20] in Figure 9) and they are transmitted via Bluetooth to remove the effect of noise from the AC power line [9], as shown in Figure 10. The output signals of the front-end amplifiers are buffered by the source follower and then the buffered signals are acquired using the analog digital converters (ADCs) on the Bluetooth module. In contrast, the output signals of the motion detectors (*V*_FL_ and *V*_FR_ in Figure 1) and the phase comparator (*V*_OL_ and *V*_OR_ in Figure 1) are digital, and hence they are obtained through GPIO of the Bluetooth module. Please note that this Bluetooth module is only used for debug purpose, that is, the module is only used to acquire the waveforms illustrated in Figure 11 and data transmission via Bluetooth is not required in practical use case. As shown in Figure 11a, when the hand moves from left to right, *V*_AL_, which denotes the output of the front-end amplifier (*V*_A_ in Figure 1) for the left electrode, first decreases because of the ESI current in the left electrode induced by the hand movement, compared with *V*_AR_, which denotes *V*_A_ for the right electrode. When *V*_AL_ and *V*_AR_ are below *V*_REF_, the outputs of the SR-latch, *V*_FL_ and *V*_FR_, are high. Because the drop in *V*_AL_ precedes that in *V*_AR_, *V*_FL_ goes high first. Consequently, *V*_OL_ becomes high, whereas V_OR_ remains low, as illustrated in the bottom waveforms in Figure 11a. In contrast, when the hand moves from right to left, the decrease in *V*_AR_ precedes that in V_AL_, as depicted in the top waveforms of Figure 11b. Thus, *V*_FR_ goes high first, which leads that *V*_OR_ to become high, while *V*_OL_ keeps low. This means that the proposed gesture recognition chip can recognize gesture correctly by observing *V*_OL_ and *V*_OR_. The ESI current depends on the thickness of the electret, as indicated in (1) and (2). This means that the thickness of the electret is one of the parameters to determine the performance of the proposed sensor. Thus, evaluating the dependence is future work. The latency of the motion detector indicated in Figure 1 is 45 ms and the total response time of the gesture recognition is 60 ms, which depends on the distance between the electrodes and the velocity of hand movement. Please note that the waveforms of *V*_AL_ and *V*_AR_ are different in Figure 11a, since the output waveform of the front-end amplifier depends on how the overlap between the hand and electrode changes, whereas the proposed sensor only uses the timing when the output voltage of the front-end amplifier changes to recognize the hand gesture and hence the difference does not affect gesture recognition.

Thereafter, we discuss the external noise from the environment and the internal noise induced by the front-end amplifier described in Section 3.1. From Figure 11, the noise observed in the output of the amplifier (*V*_AL_ and *V*_AR_) is estimated to be 10 mV_rms_, which is mainly caused by the environment (hum noise). The simulated output noise induced by the amplifier in the frequency range of 0.01–100 Hz is 1.84 mV_rms_, as shown in Figure 6, which is significantly smaller than the noise from the environment. Therefore, we can conclude that the noise induced by the amplifier can be ignored. The noise is considered as one of the reasons to worsen the stability of the proposed gesture recognition sensor. When the larger amplitude signal than the comparator input margin, which represents the voltage difference between *V*_REF_ and the common mode voltage of the front-end amplifier as illustrated in Figure 11, is induced by the noise in the input of the comparator, the hand motion is mis-detected. However, the comparator input margin in the proposed gesture recognition chip is 200 mV, while the noise amplitude is 10 mV_rms_, which is much smaller than the comparator input margin. Thus, the stability degradation caused by the noise is ignorable.

The ESI currents may be affected by humidity level of the operation environment. As reported in [9], the ESI currents mainly depend on the charge of electret and the charge of the human hand hardly affects the ESI currents. The author tested and confirmed that the proposed gesture recognition sensor can work at room temperature and moderate humidity in winter and summer, which is considered as practical operation environment. Tests under severer conditions are future work.

Finally, Table 1 compares the proposed gesture sensor with conventional sensors [4,5,6,7]. The power dissipations of the photo sensor-based [5] and capacitive sensor-based [6] techniques are large because they are active type sensors; that is, they require the light by LED and electric field, and their generation consumes significant power. In contrast, image sensor-based [4] and infrared lay-based [7] gesture sensors have been demonstrated as passive-type sensors. Their power is smaller than that of the active-type sensors [5,6], whereas bulky components such as lenses are required. The proposed gesture sensing chip consumes 406 nW at a supply voltage of 3.3 V, which is less than 1/100th of the power dissipation of conventional gesture sensors [4,5,6,7]. Furthermore, the sensing electrodes of the proposed sensor are thin and mechanically flexible, and any bulky components are not required on the electrode. On the other hand, the number of gestures that the proposed sensor can detect is smallest, as shown in Table 1. However, even such a small number of gestures is still meaningful in the target applications, such as touchless switch, described in Section 1. In addition, the number of gestures can increase if the number of electrodes increases. For example, Kurita reported that eight gestures can be detected with four electrodes [11] using electrostatic induction. Developing the sensor that can recognize more complicated gestures is future work.

## 5. Conclusions

In this paper, an ultra-low power hand gesture sensor using electrostatic induction was demonstrated. To implement gesture sensing in mobile devices, low-power gesture sensors are required because mobile devices are battery-operated. Thus, a chip for recognizing hand gestures with low power was first designed in this paper. This chip can detect the two gestures, such a hand movement from left to right and right to left, by using two electrodes, which consist of electret foils stacked on metal sheets. The measurement results revealed that the proposed gesture recognition chip consumes 406 nW, which is less than 1/100th of the power dissipation of conventional gesture sensors.

## Figures and Tables

**Table 1:** Comparison of the proposed hand gesture sensor with conventional sensors.

	ISSCC [4]	Sensors J. [5]	Sensors [6]	VLSI Symp. [7]	This Work
Sensor type	Image sensor	LED and photo sensors	Capacitive	Infrared ray	Electrostatic induction
V_DD_ (V)	0.6–1.2	1.8, 2.8	3.3 ^(*2)^	0.7, 1.4	3.3
Number of gestures	30	-	6	8	2
Distance (cm)	60	5	10	-	5
Area of sensor (cm^2^)	-	1.8	102 ^(*3)^	0.08 ^(*4)^	20 ^(*3)^
Bulky component on sensor	Required (lens)	Not required	Not required	Required (lens)	Not required
Power (μW)	184 ^(*1)^	9000	66,000 ^(*2)^	260	0.41

^(*1)^ The power consumption of image sensor is not included. ^(*2)^ The supply voltage and power dissipation are described in datasheet of MGC3130 [21]. ^(*3)^ The area of electrode. ^(*4)^ The area of lens is not included.
